# Interactive effects of water temperature and dietary protein on Nile tilapia: growth, immunity, and physiological health

**DOI:** 10.1186/s12917-024-04198-2

**Published:** 2024-08-07

**Authors:** Sara Hamed, Seham El-Kassas, Haitham G. Abo-Al-Ela, Safaa E. Abdo, Rasha A. Al Wakeel, Usama A. Abou-Ismail, Radi A. Mohamed

**Affiliations:** 1 Department of Aquaculture, Faculty of Aquatic and Fisheries Sciences, Kafrelsheikh University, Kafr El-Sheikh, Egypt; 2 Department of Animal Wealth Development, Animal, Poultry and Fish Breeding and Production, Faculty of Veterinary Medicine, Kafrelsheikh University, Kafr El-Sheikh, 33516 Egypt; 3 Department of Aquaculture, Genetics and Biotechnology, Faculty of Fish Resources, Suez University, Suez, 43221 Egypt; 4 Department of Animal Husbandry and Animal Wealth Development, Genetics and Genetic Engineering, Faculty of Veterinary Medicine, Menoufia University, Shebin El-Kom, Menoufia, 32511 Egypt; 5 Department of Animal Wealth Development, Genetics and Genetic Engineering, Faculty of Veterinary Medicine, Kafrelsheikh University, Kafr El-Sheikh, 33516 Egypt; 6 Department of Physiology, Faculty of Veterinary Medicine, Kafrelsheikh University, Kafr El-Sheikh, Egypt; 7 Department of Husbandry and Development of Animal Wealth, Faculty of Veterinary Medicine, Mansoura University, Gomhoria St, P.O. Box 35516, Mansoura, Egypt

**Keywords:** Dietary protein, Growth, Immune response, Tilapia, Water temperature

## Abstract

Optimizing fish performance depends on several factors, with dietary protein levels and rearing temperature playing important roles. In this study, Nile tilapia fingerlings (*Oreochromis niloticus*) weighing an average of 20.00 ± 1.26 g were divided into nine groups (in three replicates). Each group was subjected to different water temperatures (26 °C, 28 °C, and 30 °C) and received one of three dietary protein levels (20%, 25%, and 30%) for two months. Our findings indicate that higher temperatures, particularly at 30 °C, increased water electrical conductivity and total dissolved salts, especially noticeable in fish fed 25% or 30% crude protein (CP). Lower total ammonia nitrogen levels were observed at 28 °C with 25% CP, 30 °C with 30% CP, and 26 °C with 30% CP. Hepatic growth hormone receptor 1 and insulin-like growth factor 1 expression gradually rose with higher dietary CP percentages in fish at 26 °C but declined in those at 30 °C, albeit remaining higher than in the 28 °C groups with 25% CP. Fish at 28 °C showed the best final body weights and growth performance when fed 20% or 25% CP, with no significant difference between these groups. Hepatic leptin expression did not differ significantly among groups, but hepatic fatty acid binding protein expression notably increased in fish fed 30% CP at both 26 °C and 30 °C compared to those at 28 °C with 25% CP. Within the same temperature group, fish fed 30% CP exhibited higher globulin levels, particularly thriving at 28 °C or 30 °C. Hepatic mucin-like protein expression significantly increased across all groups, especially in fish at 30 °C with 30% CP compared to those at 28 °C with 25% CP. Hepatic lysozyme expression also increased notably in fish at 30 °C with 30% CP. Notable changes in superoxide dismutase, catalase, and glutathione peroxidase expression were observed, with the highest serum superoxide dismutase and catalase activities recorded in fish at 30 °C with 25% CP. Overall, dietary protein levels of 25% and 30%, combined with temperatures of 28 °C and 30 °C, yielded favorable outcomes, particularly favoring 28 °C with 25% protein.

**Supplementary Information:**

The online version contains supplementary material available at 10.1186/s12917-024-04198-2.

## Introduction

Aquafarming stands out as a sector with the potential to effectively address food shortages and bolster food security [1]. It provides roughly 50% of the animal protein consumed globally [2] and is projected to experience a 53% growth rate by 2030 [3]. However, global climate change poses a significant threat to aquaculture production. Specifically, global warming impacts aquaculture by altering water temperature, salinity levels, and the availability of dissolved oxygen (DO) and nutrients [4].

Fluctuations in water temperature disrupt aquatic organisms such as Nile tilapia (*Oreochromis niloticus*), inducing stress, lowering immune and antioxidant responses, increasing susceptibility to diseases, and impairing fish reproduction [2, 5]. Additionally, these temperature fluctuations hamper the growth of fish and other vital aquatic organisms such as phytoplankton and zooplankton, which are crucial components of fish nutrition [2].

These fluctuations also affect energy needs, protein and nutrient digestion, absorption efficiency, as well as stress and immune responses [6, 7]. Higher water temperatures, within physiological limits, can modulate and enhance digestive enzyme activity and protein utilization in Nile tilapia [8, 9]. However, prolonged heat stress can reduce fish growth by decreasing feed intake (FI) and nutrient utilization, including protein [10, 11]. Furthermore, heat stress triggers responses that impact protein and nutrient metabolism [10, 12].

Dietary protein levels have a significant impact on fish performance, affecting growth rates, metabolic efficiency, feed utilization, nutrient metabolism, muscle development, body composition, immune function, and reproduction activities [13, 14]. These protein requirements vary depending on factors such as fish species, growth stage, physiological activities like reproduction, and the energy content of the diet [14]. In Nile tilapia, protein needs typically range from 25 to 35%, reaching up to 50% for fry and dropping to 20% for finisher diets [15]. Protein constitutes a costly aspect of fish diets, and recent global crises have led to significant price increases, impacting profit margins and hindering aquaculture expansion [16]. Consequently, efforts have been made to reduce feed costs and optimize protein levels in diets.

However, fluctuations in water temperature can interactively alter the effects of dietary protein on fish farms, influencing fish growth and health. Therefore, when adjusting dietary protein levels in fish farms, it is crucial to consider water temperature changes due to exogenous factors such as global warming. This study aims to explore the interplay between rearing temperature and dietary protein levels, focusing on growth, FI, immune response, antioxidant capacity, blood biochemistry, and related-gene expression patterns in Nile tilapia.

## Materials and Methods

The experiment and fish management were approved and conducted according to the guidelines set by the ethical committee of Kafrelsheikh University. The authors confirm that they have adhered to EU standards for the protection of animals used for scientific purposes.

### Origin and husbandry of fish

The experiment involved 360 Nile tilapia fingerlings with an average body weight of 20.00 ± 1.26 g. These fish were obtained from a commercial hatchery in Kafrelsheikh Governorate, Egypt, and were kept in aerated plastic tanks until they were transferred to the laboratory. Upon arrival, they underwent a 2-week acclimatization period under laboratory conditions. Fish were fed a commercial tilapia diet (ALEKHWA^®^, Kafrelsheikh, Egypt) containing 25% crude protein (CP), 5.90% crude fiber, 0.39% available phosphorus, 1.1% calcium, and 2700 kcal/kg metabolizable energy. Initially, fish were fed 4% of their body mass, with feeding rates adjusted every two weeks based on changes in aquarium biomass throughout the experiment.

**Fig. 1:**
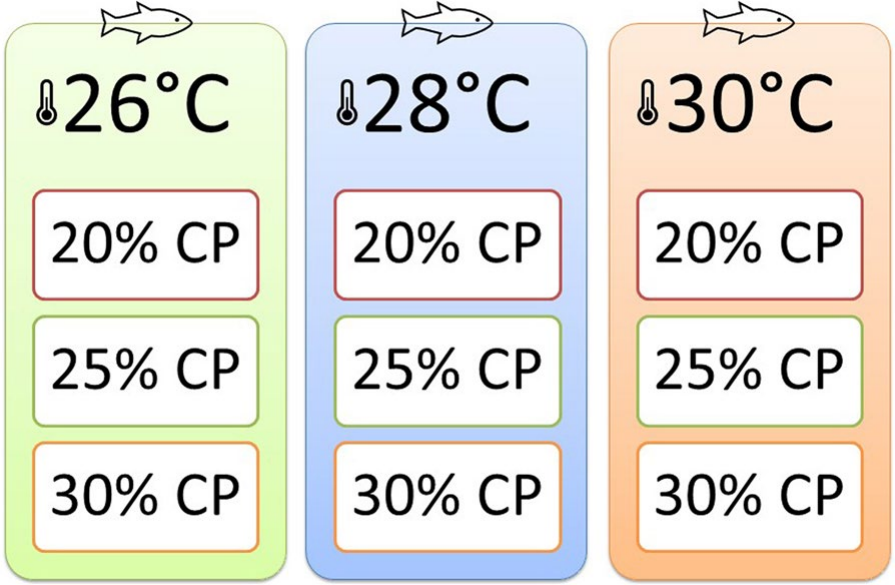
The experimental design involved rearing Nile tilapia at varying temperatures: 26ºC, 28ºC, and 30ºC. Each temperature group was further divided into three subgroups, each receiving different levels of dietary protein: 20%, 25%, and 30%

After acclimatization, the Nile tilapia were randomly distributed into 80 × 40 × 45 cm glass aquaria, with 20 fish in each aquarium. There were nine groups in total, with three subgroups each experiencing different water temperatures (26 °C, 28 °C, and 30 °C). Each temperature subgroup received three dietary protein levels (20%, 25%, and 30%), as depicted in Fig. 1. The composition of the experimental diets was adjusted by altering several components to achieve the required balanced composition. The CP percentages and metabolizable energy were set at 20% and 2650 kcal/kg, 25% and 2700 kcal/kg, and 30% and 2900 kcal/kg, respectively, as described by Hamed et al. [17] (Supplementary Table 1). The diets were formulated to meet the nutrient requirements of Nile tilapia. The experiment lasted for two months, and each treatment was replicated three times.

The aquaria water was cleaned every two days, with approximately 25% of the water replaced weekly using dechlorinated water, adjusting for the temperature of each group. The lighting cycle was maintained at 12 h light and 12 h dark throughout the experiment.

Water quality was monitored weekly at 8 am using a multi-parameter probe apparatus (HI9829-03042-HANNA^®^ instruments), assessing parameters such as DO, pH, electrical conductivity, and total dissolved solids. Total ammonia nitrogen (TAN) levels were measured using a portable colorimeter (Martini MI 405), while unionized ammonia was determined as described by Zhang et al. [18].

### Measurement and sampling

#### Growth performance

The initial body weight (IBW) was recorded at the start, and the daily FI was reported to the nearest gram. FI was calculated by subtracting the uneaten feed (collected after 20 min of feeding, then dried and weighed) from the amount offered. Two months into the experiment, the final body weight (FBW) was measured by weighing the water-dried fish. Additionally, body thickness, width, and length were meticulously measured using the method outlined by Bagenal [19].

$$\text{Body weight gain }\left(\text{BWG};\text{g}\right)=\text{IBW}-\text{ FBW}$$

$$\text{Feed conversion ratio }(\text{FCR}) =\frac{\text{FI }(\text{g})}{\text{Weight gain }(\text{WG};\text{ g})}$$

$$\text{Specific growth rate }(\text{SGR};\text{\%})=\frac{\text{ln}\left(\text{FBW}(\text{g})\right)-\text{ ln}\left(\text{IBW }\left(\text{g}\right)\right)\times 100}{\text{t }(\text{in days})}$$

#### Sampling

Two months into the experiment, we randomly collected three fish per aquarium, resulting in a total of nine samples per treatment. The fish were sedated with tricaine methanesulfonate (MS-222) at a concentration of 35 mg/L. Blood samples were promptly taken from the tail vein using a sterile syringe, which were then used for serum separation and preserved at –20ºC. Additionally, liver samples were collected from the sampled tilapias, immediately frozen in liquid nitrogen, and stored at –80 °C for RNA extraction.

### Blood biochemical profiling

Glucose, total cholesterol, triglycerides, total blood protein, and albumin were measured in the serum samples using commercial kits from Biodiagnostic Co., Egypt. Serum globulin levels were calculated by subtracting albumin levels from total protein levels. Liver function enzymes, including aspartate aminotransferase (AST) and alanine aminotransferase (ALT), were also measured using Biodiagnostic Co. kits (Egypt). Additionally, catalase (CAT), superoxide dismutase (SOD), and malondialdehyde (MDA) levels were determined at wavelengths of 510 nm, 560 nm, and 534 nm, respectively, with Biodiagnostic Co. kits (Egypt). Serum immunoglobulin M (IgM) and lysozyme (LYZ) were measured at 450 nm using an ELISA kit from Cusabio Biotech Co. Ltd., Wuhan, China, following their protocol.

### Real-time PCR analysis of selected genes regulating growth, feed intake, lipid metabolism, antioxidants, and innate immunity

RNA was extracted from liver samples (*n* = 9 per treatment) using TRIzol™ Reagent from Thermo Fisher Scientific Inc, following the manufacturer’s protocol. The integrity of the RNA was validated by electrophoresis in a 2% agarose gel, and its purity and concentration were assessed using a NanoDrop. Subsequently, cDNA was synthesized from 2 µg of the extracted RNA using specific cDNA kits from iNtRON Biotechnology.

Real-time PCR was conducted for selected growth genes, including growth hormone receptor 1 (*ghr1*) and insulin-like growth factor 1 (*igf1*), the FI gene leptin, the fatty acid binding protein (*fabp*), and antioxidant genes such as superoxide dismutase (*sod*), catalase (*cat*), and glutathione peroxidase (*gpx*), along with innate immunity genes such as mucin-like protein (*muc*), oligo-peptide transporter 1 (*pept1*), and lysozyme (*lyz*). The *β*-actin housekeeping gene served as an internal reference gene. Specific primers are listed in Table 1. PCR reactions were run in duplicate using 0.5 μM forward and reverse primers, 2 μL of cDNA, and SensiFast™ SYBR Lo-Rox master mix from Bioline (United Kingdom).

**Table 1:** The sequences of primers used in the study

Gene	Primer	GenBank accession NO	Reference
*β-actin*	F: CAGCAAGCAGGAGTACGATGAG R: TGTGTGGTGTGTGGTTGTTTTG	XM_003455949.2	Abo-Al-Ela et al. [20] and El-Kassas et al. [16]
*ghr1*	F: CAGACTTCTACGCTCAGGTC R: CTGGATTCTGAGTTGCTGTC	MW509678.1	
*igf1*	F: GTTTGTCTGTGGAGAGCGAGG R: GAAGCAGCACTCGTCCACG	NM_001279503.1	
*fabp*	F: CAAGCCCACCACCATCATCT R: TTCCCGTCCTCTATCGTGACA	XM_003444047.5	
leptin	F: AGGCTGGACAAAGACGTACA R: AACCGTTCAAGACCGTCTCT	NM_001301050.1	
*muc*	F: TGCCCAGGAGGTAGATATGC R: TACAGCATGAGCAGGAATGC	XM_005466350.2	Aanyu et al. [21]
*pept1*	F: CAAAGCACTGGTGAAGGTCC R: CACTGCGTCAAACATGGTGA	XM_013271589.3	
*lyz*	F: AAGGGAAGCAGCAGCAGTTGTG R: CGTCCATGCCGTTAGCCTTGAG	XM_003460550.2	Esam et al. [22]
*sod*	F: CATGCTTTTGGAGACAACAC R: ACCTTCTCGTGGATCACCAT	XM_003446807.5	El-Haroun et al. [23]
*cat*	F: CCCAGCTCTTCATCCAGAAAC R: GCCTCCGCATTGTACTTCTT	JF801726.1	Abdo et al. [24]
*gpx*	F: CCAAGAGAACTGCAAGAACGA R: CAGGACACGTCATTCCTACAC	DQ355022.1	El-Kassas et al. [25]

*igf1 *insulin-like growth factor 1, *ghr* growth hormone receptor, *muc* mucin-like protein, *pept1* oligo-peptide transporter 1, *fabp* fatty acid binding protein, *sod* superoxide dismutase, *cat* catalase, *gpx* glutathione peroxidase, *lyz* lysozyme

The cycling conditions were set on a Stratagene MX300P real-time PCR system (Agilent Technologies) as follows: initial denaturation at 95 °C for 15 min, followed by 40 cycles of 95 °C for 15 s, annealing at 60 °C for 1 min, and 72 °C for 30 s. Dissociation curves were analyzed to ensure the presence of a single peak at a specific melting temperature, confirming specific amplification of PCR products. Relative gene expression was calculated using the 2^−ΔΔCt^ method. Tilapias farmed in water at 28 °C and receiving 25% CP were considered as the control group.

### Statistical analysis

The results data were analyzed using the generalized linear model (GLM) procedure in IBM SPSS Statistics (version 22, SPSS Inc., IL, USA), with significance set at *P* < 0.05. Normality and homogeneity of variance were checked using the Shapiro–Wilk and Levene tests, respectively. Two-way ANOVA was employed to examine the effects of water temperature, dietary protein percentages, and their interactions. Tukey’s HSD test was used for multiple comparisons to assess statistical significance. The results were presented as mean ± SEM, and figures were created using GraphPad Prism 9 (La Jolla, California, USA).

## Results

### Changes in water quality parameters

The water quality parameters exhibited variations among the experimental groups (Table 2). Specifically, water electrical conductivity (EC) and total dissolved salts (TDS) significantly increased in the group reared at 28 °C and given 20% CP, as well as in those reared at 30 °C and given 25% CP or 30% CP. These changes were attributed to temperature fluctuations rather than changes in CP percentage. However, CP percentage did have a significant impact on these parameters in the group reared at 30 °C and given 20% CP. Increasing the CP percentage significantly raised the levels of TAN, with temperature having a minor effect. However, both temperature and CP percentage, along with their interaction, showed significant effects on TAN levels. The variations in temperature and CP percentage also had an interaction effect on DO, with lower values observed at 28 °C with 25% CP, 30 °C with 30% CP, and 26 °C with 30% CP.

**Table 2:** Water quality parameters of the different groups raised under varied temperatures and dietary protein percentages

	**EC (μS/L)**	**TDS (mg/L)**	**Water pH**	**TAN (mg/L)**	**DO (mg/L)**
**26ºC**					
**20% CP**	663.00 ± 22.02^**Ba**^	331.57 ± 10.99^**Ba**^	7.57 ± 0.07^**Aa**^	0.59 ± 0.10^**Ab**^	4.67 ± 0.26^**Aa**^
**25% CP**	653.00 ± 13.12^**Ba**^	324.79 ± 6.29^**Ba**^	7.48 ± 0.08^**Aa**^	0.18 ± 0.03^**Ac**^	4.63 ± 0.23^**ABa**^
**30% CP**	696.36 ± 19.52^**Ba**^	349.21 ± 9.73^**Ba**^	7.40 ± 0.09^**Aa**^	1.20 ± 0.06^**Aa**^	4.40 ± 0.26^**Ba**^
**28ºC**					
**20% CP**	727.21 ± 20.40^**Aa**^	363.57 ± 10.23^**Aa**^	7.55 ± 0.08^**Aa**^	0.060 ± 0.02^**Bb**^	5.10 ± 0.15^**Aa**^
**25% CP**	705.86 ± 17.91^**Ba**^	353.00 ± 8.98^**ABa**^	7.45 ± 0.07^**Aa**^	0.120 ± 0.03^**Ab**^	4.10 ± 0.31^**Bb**^
**30% CP**	683.64 ± 17.24^**Ba**^	341.79 ± 8.56^**Ba**^	7.51 ± 0.08^**Aa**^	0.480 ± 0.03^**Ba**^	5.27 ± 0.14^**Aa**^
**30ºC**					
**20% CP**	719.36 ± 15.04^**Ba**^	359.64 ± 7.49^**ABb**^	7.60 ± 0.06^**Aa**^	0.11 ± 0.03^**Bb**^	5.11 ± 0.12^**Aa**^
**25% CP**	742.14 ± 16.26^**Aa**^	370.86 ± 8.03^**Aab**^	7.43 ± 0.07^**Aab**^	0.10 ± 0.02^**Ab**^	5.30 ± 0.37^**Aa**^
**30% CP**	779.71 ± 19.02^**Aa**^	397.86 ± 9.26^**Aa**^	7.34 ± 0.07^**Ab**^	0.48 ± 0.04^**Ba**^	4.32 ± 0.13^**Bb**^
***P* values**					
**Temperature**	< 0.001	< 0.001	0.744	< 0.001	0.186
**D. Protein%**	0.36	0.146	0.033	< 0.001	0.213
**Interaction**^a^	0.051	0.016	0.688	< 0.001	< 0.001

The raw data were analyzed from Hamedet al. [17]. Different letters donate for statistical significance at *P* < 0.05. Uppercase letters signify significance due to water temperature, while lowercase letters signify significance due to dietary crude protein (CP) percentages

*EC* Water electrical conductivity, *TDS* Total dissolved salts, *pH* Water pH, *DO *Dissolved oxygen, *TAN *Total ammonia nitrogen

^a^Donates the water temperature x dietary protein percentage interaction

### Growth performance

Moderate FBW was observed in fish reared at 26 °C and given 30% CP and at 28 °C and given 20% CP (Table 3). The highest FBW was recorded in fish reared at 30 °C and given 25% CP, although there were no significant changes among the other groups. This trend was also reflected in total weight gain (TWG). FI did not show significant changes across the groups. Changes in CP percentage significantly affected the FCR, with the lowest values observed in fish given 25% and 30% CP (Table 3). A similar pattern was observed in SGR, with the highest values seen in fish given 25% and 30% CP. However, there was no significant difference between those reared at 28 °C and given 20% or 25% CP. Temperature had a notable impact on final body thickness (FT), while CP percentage had little to no effect. Fish reared at 28 °C and 30 °C showed the highest increases in FT, except for those reared at 28 °C with 20% CP (Table 3).

**Table 3:** Growth performance of the different groups raised under varied temperatures and dietary protein percentages

	**IBW (g)**	**FBW (g)**	**FI (g)**	**TWG (g)**	**FCR**	**SGR (%)**	**FL (cm)**	**FW (cm)**	**FT (mm)**
**26ºC**									
**20% CP**	21.3 ± 0.057	44.52 ± 1.35^**Bb**^	59.52 ± 0.0	22.22 ± 1.35^**Bb**^	1.34 ± 0.035^**Aa**^	3.70 ± .022^**Bb**^	14.02 ± 0.42	4.39 ± 0.18	20.74 ± 0.93^**Bb**^
**25% CP**	22.24 ± 0.124	51.92 ± 2.30^**Ba**^	56.4 ± 0.0	29.68 ± 2.28^**Ba**^	1.10 ± 0.049^**Ab**^	4.95 ± .38^**Ba**^	14.64 ± 0.64	4.83 ± 0.24	20.78 ± 0.86^**Bb**^
**30% CP**	22.20 ± 0.042	55.16 ± 2.54^**Ba**^	56.88 ± 0.0	32.96 ± 2.255^**Ba**^	1.05 ± 0.05^**Ab**^	5.49 ± 0.43^**Ba**^	14.80 ± 0.48	4.70 ± 0.14	22.62 ± 0.68^**Ba**^
**28ºC**									
**20% CP**	22.3 ± 0.5	54.96 ± 2.44^**ABa**^	60.52 ± 0.0	32.66 ± 2.44^**ABa**^	1.12 ± 0.05^**Aa**^	5.44 ± 0.41^**Ab**^	14.53 ± 0.47	4.74 ± 0.15	20.51 ± 1.29^**Bb**^
**25% CP**	21 ± 0.32	52.74 ± 2.7^**ABa**^	57.4 ± 0.0	30.74 ± 2.7^**ABa**^	1.11 ± 0.5^**Aa**^	5.12 ± 0.45^**Ab**^	14.71 ± 0.50	4.53 ± 0.16	22.14 ± 0.78^**Aa**^
**30% CP**	22.6 ± 0.21	60.58 ± 5.19^**ABa**^	61.24 ± 0.0	37.98 ± 5.91^**ABa**^	1.05 ± 0.07^**Aab**^	6.31 ± 0.86^**Aa**^	14.66 ± 0.67	4.61 ± 0.22	23.26 ± 1.11^**Aa**^
**30ºC**									
**20% CP**	22.3 ± 0.31	49.58 ± 2.03^**Ab**^	60.52 ± 0.0	27.29 ± 2.03^**Ab**^	1.235 ± 0.05^**Aa**^	4.57 ± 0.34^**Ab**^	14.43 ± 0.53	4.30 ± 0.21	24.35 ± 0.55^**Aa**^
**25% CP**	22 ± 0.24	65.96 ± 4.12^**Aa**^	59.8 ± 0.0	43.96 ± 4.12^**Aa**^	0.93 ± 0.061^**Bb**^	7.33 ± 0.69^**Aa**^	15.23 ± 0.61	4.69 ± 0.29	24.31 ± 1.17^**Aa**^
**30% CP**	22 ± 0.74	61.1 ± 3.57^**Aa**^	59.8 ± 0.0	39.1 ± 3.57^**Aa**^	1.01 ± 0.066^**Ab**^	6.52 ± 0.59^**Aa**^	15.43 ± 0.37	4.91 ± 0.21	25.42 ± 1.08^**Aa**^
**P values**									
**Temperature**	0.541	0.006	0.635	0.005	0.057	0.005	0.430	0.996	< 0.001
**CP%**	0.897	0.001	0.521	0.001	< 0.001	0.001	0.288	0.258	0.052
**Interaction**^a^	0.974	0.063	0.237	0.065	0.056	0.065	0.943	0.329	0.870

The raw data were analyzed from Hamedet al. [17]. Different letters donate for statistical significance at *P* < 0.05. Uppercase letters signify significance due to water temperature, while lowercase letters signify significance due to dietary crude protein (CP) percentages

*IBW* Initial body weight, *FBW* Final body weight, *FI *Feed intake, *TWG *Total weight gain, *FCR *Feed conversion ratio, *SGR *Specific growth rate, *FL *Final body length, *FW *Final body width, *FT *Final body thickness.

^a^Donates the water temperature x dietary protein percentage interaction

### Biochemical serum profiling

The temperature had an impact on the glucose levels of the groups, with the lowest levels observed in fish raised at 28 °C and given either 25% CP or 30% CP (Table 4). Total protein levels showed similar trends in fish given 20% CP or 25% CP and raised at 26 °C or 30 °C. Clearly, temperature and the interaction between temperature and CP percentage significantly affected total protein levels. This change was mainly due to fluctuations in globulin levels. Fish raised at 28 °C and those raised at 30 °C and given 30% CP had the highest globulin levels, with no significant difference between them (Table 4).

**Table 4:** Serum biochemical profile of the different groups raised under varied temperatures and dietary protein percentages

	**Glucose (mg/dL)**	**Total protein (g/dL)**	**Globulin (g/dL)**	**Albumin (g/dL)**	**AST (U/L)**	**ALT (U/L)**
**26ºC**						
**20% CP**	12.78 ± 0.12^**Ab**^	3.23 ± 0.02^**Bb**^	1.23 ± 0.01^**Bb**^	1.99 ± 0.01^**Bb**^	96.39 ± 0.44^**Aa**^	7.30 ± 0.01^**Bb**^
**25% CP**	13.81 ± 0.14^**Aa**^	3.37 ± 0.05^**Ba**^	1.23 ± 0.03^**Bb**^	2.14 ± 0.02^**Aa**^	76.83 ± 1.95^**Bc**^	7.55 ± 0.04^**Aa**^
**30% CP**	13.88 ± 0.07^**Aa**^	3.44 ± 0.01^**Aa**^	1.38 ± 0.03^**Ba**^	2.06 ± 0.04^**Aa**^	86.50 ± 0.61 ^**Ab**^	7.52 ± 0.07^**Aa**^
**28ºC**						
**20% CP**	12.85 ± 0.74^**Aa**^	3.67 ± 0.03^**Aa**^	1.46 ± 0.04^**Ab**^	2.21 ± 0.01^**Aa**^	82.42 ± 0.66 ^**Ba**^	7.43 ± 0.03^**Ab**^
**25% CP**	11.02 ± 0.25^**Bb**^	3.54 ± 0.01^**Aa**^	1.39 ± 0.8^**Ab**^	2.15 ± 0.10^**Aa**^	80.72 ± 0.22 ^**Aa**^	7.63 ± 0.10^**Aa**^
**30% CP**	12.21 ± 0.20^**Ba**^	3.44 ± 0.01^**Aa**^	1.52 ± 0.04^**Aa**^	1.92 ± 0.02^**Bb**^	79.95 ± 0.35 ^**Bb**^	7.28 ± 0.04^**Bb**^
**30ºC**						
**20% CP**	13.22 ± 0.19^**Aa**^	3.39 ± 0.06^**Ba**^	1.24 ± 0.05 ^**Ba**^	2.15 ± 0.01^**Aa**^	76.07 ± 0.80 ^**Bb**^	7.32 ± 0.04^**Ba**^
**25% CP**	12.99 ± 0.027^**Ab**^	3.29 ± 0.15^**Bb**^	1.25 ± 0.02^**Ba**^	2.04 ± 0.04^**Aa**^	80.27 ± 0.09^**Aa**^	7.16 ± 0.04^**Bb**^
**30% CP**	13.77 ± 0.17^**Aa**^	3.51 ± 0.06^**Aa**^	1.63 ± 0.08^**Ab**^	1.88 ± 0.02^**Aa**^	79.25 ± 1.54^**Bb**^	7.29 ± 0.06^**Bb**^
***P* values**						
**Temperature**	< 0.001	< 0.001	0.001	0.128	< 0.001	0.001
**CP %**	0.049	0.116	< 0.001	< 0.001	< 0.001	0.111
**Interaction**^a^	0.004	< 0.001	0.032	0.002	< 0.001	0.001

Different letters donate for statistical significance at *P* < 0.05. Uppercase letters signify significance due to water temperature, while lowercase letters signify significance due to dietary crude protein (CP) percentages

*AST *Aspartate aminotransferase, *ALT* Alanine aminotransferase

^a^Donates the water temperature x dietary protein percentage interaction

However, CP percentages and their interaction with temperature influenced the albumin levels of the studied groups. The lowest AST values were found in fish raised at 26 °C and given 25% CP, as well as in those raised at 28 °C or 30 °C and given either 20% CP or 30% CP (Table 4). ALT levels were lowest in fish raised at 26 °C and given 20% CP, as well as in those raised at 28 °C and given 30% CP, or raised at 30 °C, while other groups showed slight increases, influenced by temperature and the interaction between temperature and CP percentage (Table 4).

Cholesterol levels did not significantly vary across the groups, whereas triglycerides changed in response to rearing temperature (Table 5). Specifically, the lowest significant values of triglycerides were seen in fish raised at 28 °C and given either 20% CP or 25% CP.

**Table 5:** Serum levels of cholesterol and triglycerides and activities of antioxidant and immune enzymes of the different groups raised under varied temperatures and dietary protein percentages

	**Cholesterol (mg/dL)**		**Triglycerides (mg/dL)**	**MDA (nmol/g)**	**IgM (µg/mL)**	**CAT (U/g)**	**SOD (U/g)**	**LYZ (µg/mL)**
**26ºC**								
**20% CP**	75.60 ± 2.46		92.85 ± 3.69^**ABb**^	17.87 ± 0.36^**Aa**^	5.31 ± 0.02^**b**^	17.34 ± 0.03^**Aab**^	9.84 ± 0.01^**Ba**^	10.21 ± 0.07^**Aa**^
**25% CP**	82.20 ± 1.13		111.91 ± 11.75^**Aa**^	17.63 ± 0.41^**Aa**^	5.13 ± 0.02^**b**^	17.44 ± 0.15^**a**^	9.84 ± 0.01^**Ba**^	10.32 ± 0.05^**Aa**^
**30% CP**	81.28 ± 0.61		105.00 ± 3.03^**Aa**^	15.95 ± 0.55^**Bb**^	5.56 ± 0.075^**a**^	17.16 ± 0.23^**b**^	9.97 ± 0.02^**Aa**^	9.51 ± 0.08^**Bb**^
**28ºC**								
**20% CP**	81.75 ± 0.86		84.75 ± 0.23^**Bb**^	15.89 ± 0.51^**Bb**^	5.08 ± 0.02^**b**^	17.16 ± 0.10^**b**^	10.03 ± 0.02^**Aa**^	10.77 ± 0.18^**Aa**^
**25% CP**	80.84 ± 0.26		85.40 ± 1.70^**Bb**^	16.01 ± 0.47^**Ba**^	5.47 ± 0.06^**a**^	17.10 ± 0.15^**b**^	9.99 ± 0.07^**Bb**^	10.06 ± 0.12^**Aa**^
**30% CP**	83.79 ± 4.35		108.96 ± 8.4^**Aa**^	16.39 ± 0.31^**Aa**^	5.28 ± 0.04^**b**^	17.91 ± 0.017^**a**^	9.83 ± 0.02^**Ab**^	10.22 ± 0.19^**Aa**^
**30ºC**								
**20% CP**	78.48 ± 0.69		107.41 ± 9.90^**Aa**^	17.81 ± 0.35^**Aa**^	5.50 ± 0.06^**a**^	16.97 ± 0.05^**Bb**^	10.18 ± 0.09^**Aa**^	9.99 ± 0.014^**Bb**^
**25% CP**	81.12 ± 0.97		128.58 ± 2.42^**Aa**^	17.99 ± 0.05^**Aa**^	5.19 ± 0.02^**b**^	17.29 ± 0.03^**a**^	10.36 ± 0.03^**Aa**^	10.82 ± 0.18^**Aa**^
**30% CP**	79.09 ± 1.48		104.85 ± 8.76^**Aa**^	17.33 ± 0.20^**Aa**^	5.24 ± 0.03^**b**^	17.29 ± 0.01^**a**^	9.94 ± 0.04^**Ab**^	10.28 ± 0.04^**Aa**^
***P* values**								
**Temperature**		0.213	0.010	0.001	0.298	0.137	< 0.001	0.002
**CP %**		0.153	0.068	0.104	0.053	0.020	0.002	0.001
**Interaction**^a^		0.316	0.057	0.062	< 0.001	0.002	< 0.001	< 0.001

Different letters donate for statistical significance at *P* < 0.05. Uppercase letters signify significance due to water temperature, while lowercase letters signify significance due to dietary crude protein (CP) percentages

*MDA *Malondialdehyde, *IgM *Immunoglobulin M, *CAT* Catalase, *SOD* Sodium oxide dismutase, *LYZ* Lysozyme

^a^Donates the water temperature x dietary protein percentage interaction.

On the immune level, serum MDA levels were lower in fish raised at 26 °C and fed 30% CP, as well as in those raised at 28 °C and fed either 20% or 25% CP (Table 5). It is notable that MDA levels were particularly lower in fish raised at 28 °C. A slight interaction between temperature and CP percentage was observed to influence serum IgM levels. Temperature seemed to have less or no effect, whereas CP percentage and their interaction had an impact on serum CAT levels (Table 5). Specifically, fish raised at 28 °C and fed 30% CP exhibited the highest serum CAT level. Both temperature and CP percentage, along with their interaction, showed a significant impact on serum levels of SOD and LYZ (Table 5). The most significant values were observed in fish raised at 28 °C with either 25% or 30% CP. However, the highest levels of SOD and LYZ were recorded in fish raised at 30 °C with 25% CP.

### Relative changes in gene expression of relevant gene regulating growth, and immune and oxidative responses

**Fig. 2:**
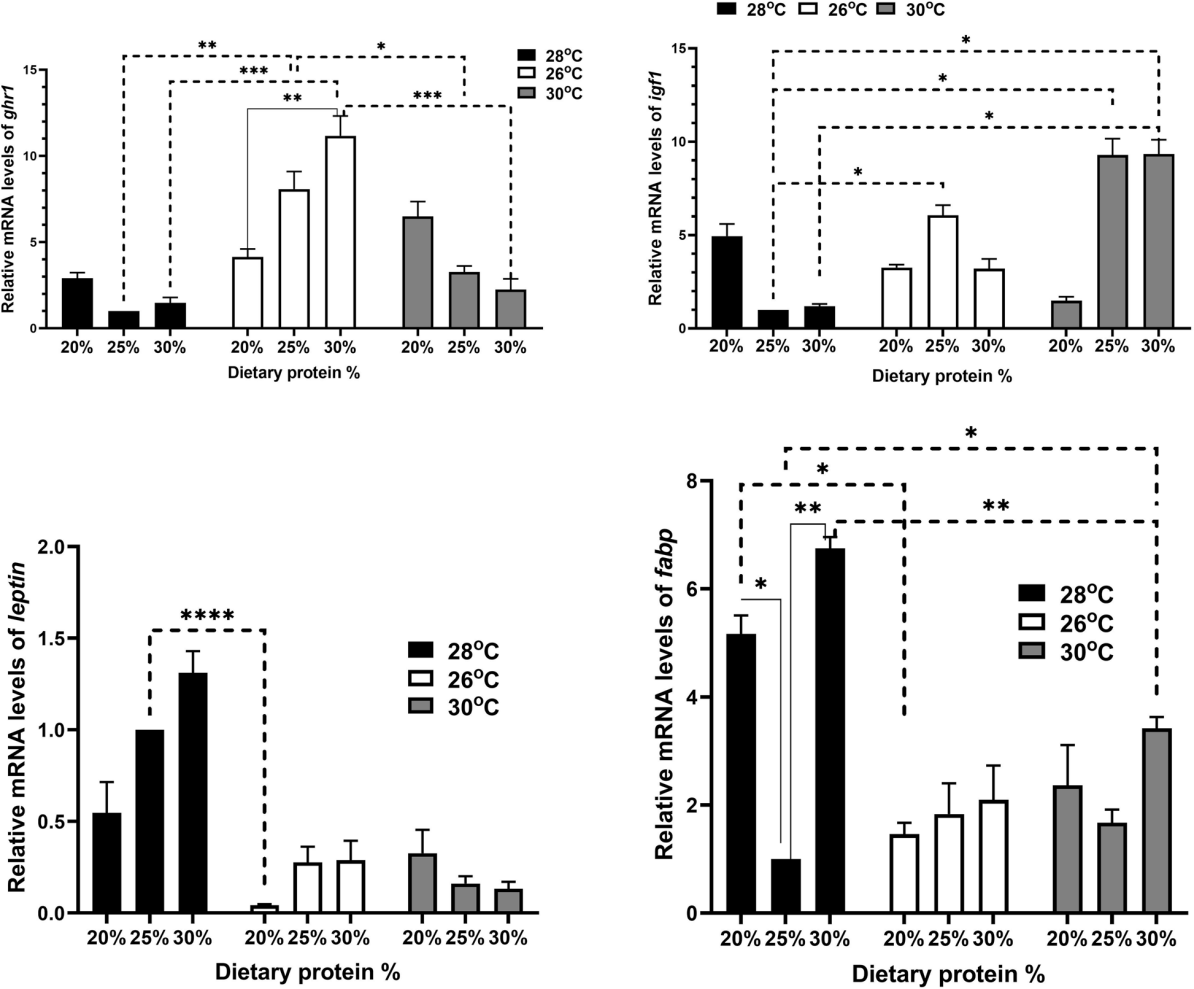
Relative gene expression results for growth hormone receptor 1 (*ghr1*), insulin-like growth factor 1 (*igf1*), leptin, and fatty acid binding protein (*fabp*) in Nile tilapia (*Oreochromis niloticus*) fingerlings raised under three water temperatures (26ºC, 28ºC, and 30ºC) and three different levels of dietary protein (20%, 25%, and 30%) at each temperature for a duration of two month. The fish reared at 28 °C with 25% crude protein (CP) served as the control, analyzed using the 2^−ΔΔCt^ method. Data are presented as mean ± SEM

The hepatic *ghr1* expression increased gradually with higher dietary CP percentages, especially in fish raised at 26 °C. Notably, there was significant upregulation in the groups raised at 26 °C and receiving 25% or 30% CP compared to those raised at 28 °C with the same dietary CP levels (Fig. 2). However, as the dietary CP percentage increased, there was a corresponding downregulation of *ghr1* expression in the group raised at 30 °C, although it remained higher than levels observed in fish raised at 28 °C. Regarding *igf1* expression, there was a noticeable increase, especially in fish raised at 30 °C and given 25% or 30% CP, as well as those raised at 26 °C and given 25% CP.

Leptin expression in the liver showed a non-statistically significant increase in fish raised at 28 °C and given 30% CP. Conversely, it was downregulated in the other groups, with significance observed only in the 26 °C group with 20% CP compared to fish at 28 °C with 25% CP (Fig. 2). On the other hand, the expression of *fabp* increased in all groups compared to fish at 28 °C with 25% CP (Fig. 2). Significant increases were noted in fish at 28 °C with 20% or 30% CP and those at 30 °C with 30% CP.

**Fig. 3:**
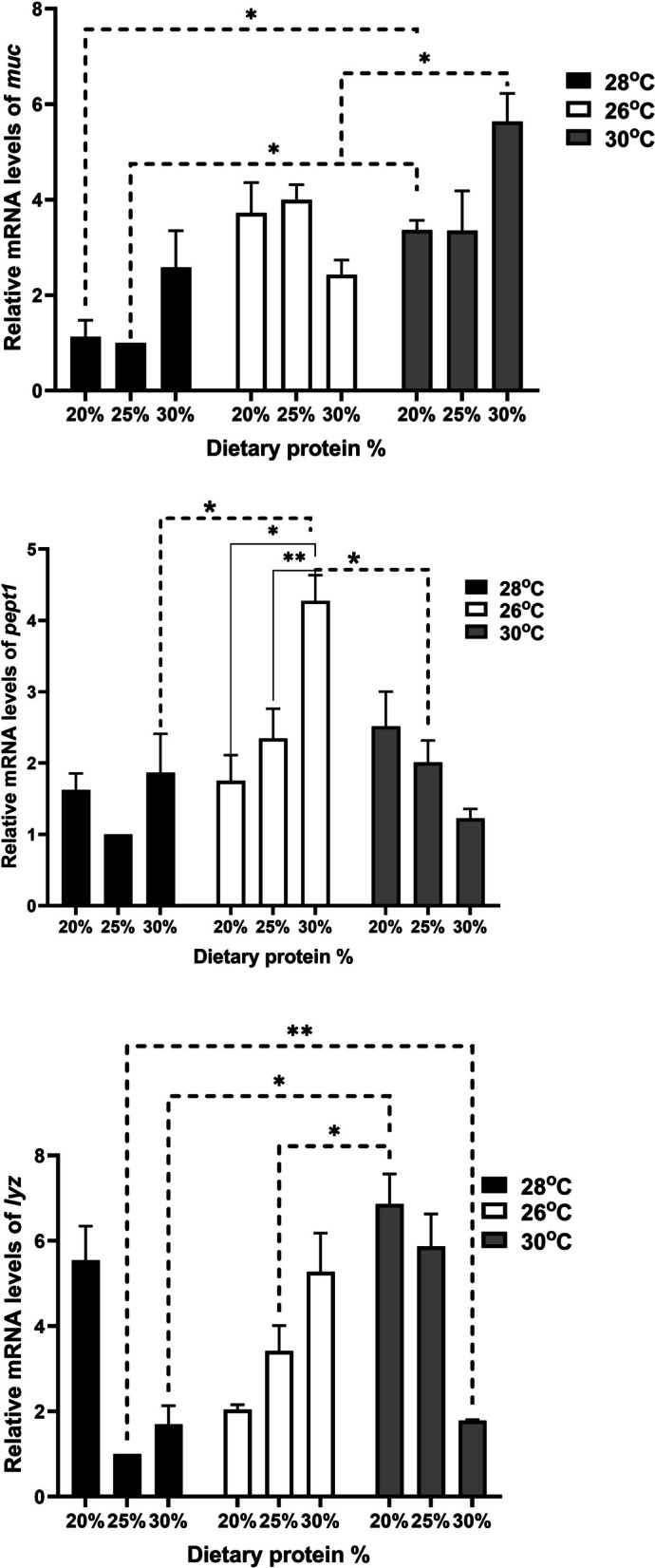
Relative gene expression results for mucin-like protein (*muc*), oligo-peptide transporter 1 (*pept1*), and lysozyme (*lyz*) in Nile tilapia (*Oreochromis niloticus*) fingerlings raised under three water temperatures (26ºC, 28ºC, and 30ºC) and three different levels of dietary protein (20%, 25%, and 30%) at each temperature for a duration of two month. The fish reared at 28 °C with 25% crude protein (CP) served as the control, analyzed using the 2^−ΔΔCt^ method. Data are presented as mean ± SEM

Hepatic expression of *muc* increased across all groups, with a significant rise noted in fish at 30 °C with 30% CP compared to those at 28 °C with 25% CP (Fig. 3). A similar trend was seen in *pept1* and *lyz*, although *lyz* expression was notably significant in fish at 30 °C with 30% CP (Fig. 3).

**Fig. 4:**
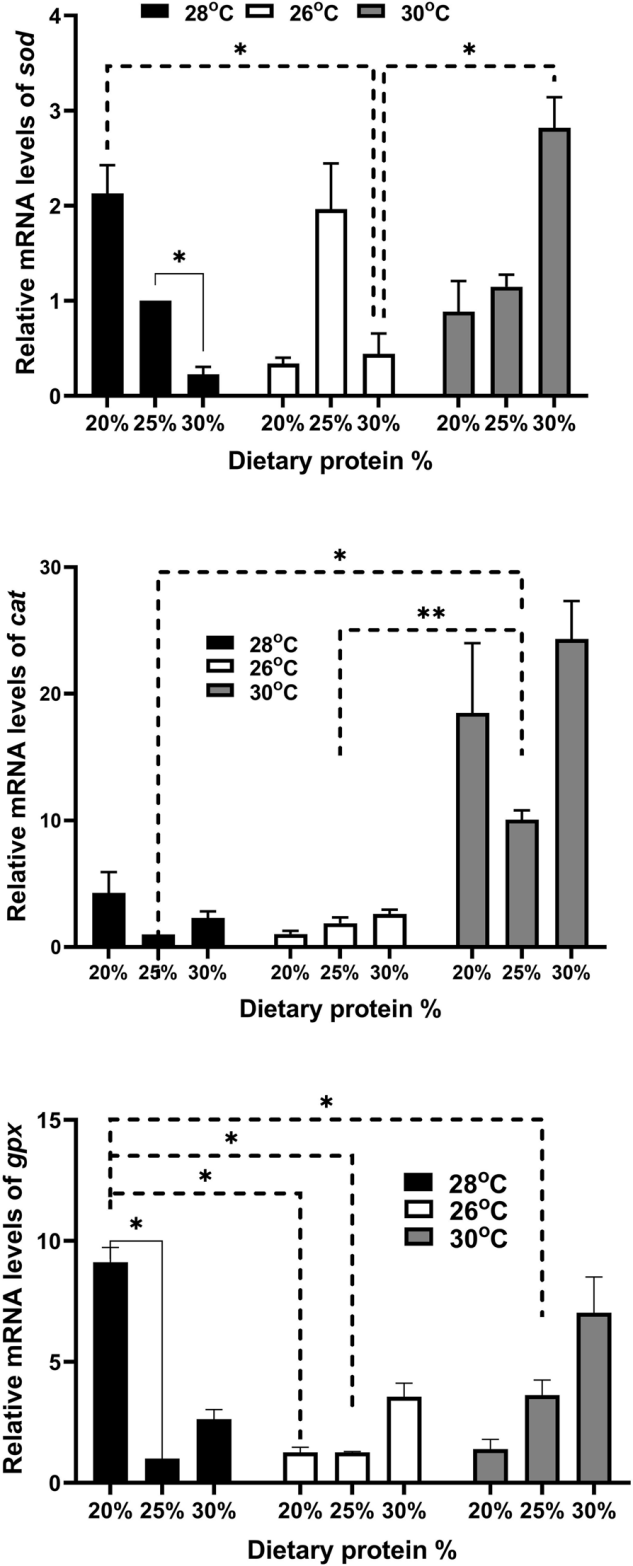
Relative gene expression results for superoxide dismutase (*sod*), catalase (*cat*), and glutathione peroxidase (*gpx*) in Nile tilapia (*Oreochromis niloticus*) fingerlings raised under three water temperatures (26ºC, 28ºC, and 30ºC) and three different levels of dietary protein (20%, 25%, and 30%) at each temperature for a duration of two month. The fish reared at 28 °C with 25% crude protein (CP) served as the control, analyzed using the 2^−ΔΔCt^ method. Data are presented as mean ± SEM

The expression of *sod* increased in fish raised at 28 °C with 20% CP, 26 °C with 25% CP, and 30 °C with 25% or 30% CP. Conversely, the remaining groups showed downregulation compared to those at 28 °C with 25% CP (Fig. 4). These changes were mostly not significant, except for the notable downregulation observed in fish at 28 °C with 30% CP, as well as the upregulation seen in fish at 26 °C with 30% CP compared to those at 30 °C with 30% CP.

Regarding the hepatic expression of *cat*, there was a range from slight to marked upregulation in fish raised at 30 °C, with significant changes noted in fish at 30 °C with 25% CP (Fig. 4). Additionally, the hepatic expression of *gpx* also increased, with a notable increase observed in fish at 28 °C with 20% CP (Fig. 4).

## Discussion

The current study observed a wide range of changes in various parameters, notably affected by rearing water temperature, variations in dietary protein percentage, and their interaction. Among these parameters, water quality stood out for its significant impact on aquatic animal health and production. Water parameters play a crucial role in influencing immune and antioxidant responses as well as fish growth. Rearing conditions like stocking density and temperature can regulate water ammonia levels and other physiological parameters affecting growth factors (e.g., circulating levels of triiodothyronine (T_3_) or blood profiling), which in turn can either suppress or enhance final body weights [26–28].

The findings indicated that increasing temperature led to higher levels of EC and TDS, particularly in fish given 25% CP or 30% CP and reared at 30 °C. TDS has been associated with changes in temperature and EC [29]. While temperature correlates with DO (especially in polluted or poor water quality) and pH [30], the temperature changes in our study didn’t significantly affect these parameters, possibly due to the narrow temperature range or controlled rearing system. Similarly, increasing CP% resulted in higher TAN levels, with an interaction effect between temperature and dietary protein percentage. Lower values were observed at 28 °C with 25% CP, 30 °C with 30% CP, and 26 °C with 30% CP. TAN and free ammonia nitrogen concentrations are positively correlated with dietary protein levels [31–33]. Elevated ammonia levels disrupt normal physiological balance, hindering growth and increasing disease susceptibility [34]. Thus, maintaining a balance between CP% and water ammonia levels is crucial for optimal growth and health outcomes.

Growth is significantly influenced by CP% and temperature, as demonstrated in our findings and those of others [35–37]. However, protein requirements can vary based on factors such as fish size and age, feeding habits, water temperature, and salinity [38]. Among these factors, temperature plays a crucial role. Studies have shown that when temperatures exceed optimal conditions, there is a decrease in serum glucose and triglyceride levels [37]. Additionally, the activities of intestinal trypsin, lipase, and amylase, along with hepatic lysozyme, are reduced. This leads to downregulation of *igf1* in the liver and an increase in oxidative stress in rainbow trout (*Oncorhynchus mykiss*) [37].

The hypothalamic–pituitary–somatotropic axis is governed by various molecules, with Igf1 and Gh playing critical roles [39, 40]. Alterations in upbringing or environmental factors, such as temperature, significantly impact this physiological axis. These changes lead to a series of modifications in pathways that influence body growth and homeostasis [40]. The expression of *igf1* and *ghr* is particularly linked to feeding patterns [41]. IGF1 is mainly produced by the liver when stimulated by GH [42]. Interestingly, reduced protein intake in animals led to lower insulin and IGF1 levels while leaving GH levels unaffected. Furthermore, there was a downregulation in GHR expression in response to decreased insulin, possibly influenced by decreased calcium blood levels due to reduced dietary protein [43]. Similarly, the current findings indicated a gradual increase in hepatic *ghr1* expression with rising dietary CP percentages in fish raised at 26 °C. Conversely, this trend reversed with a gradual decrease in groups raised at 30 °C, although values remained higher compared to those raised at 28 °C and given 25% CP. In these same groups, *igf1* expression was notably elevated in the liver, indicating a positive correlation. Notably, serum glucose levels were affected by temperature but not dietary protein alteration. These findings suggest that temperature could modulate metabolic and growth-related pathways in response to dietary components. Once again, this suggests a potential interaction between water temperature and dietary protein percentage.

These results align with the records for FBW. The best values were observed in fish raised at 28 °C with both 20% CP and 25% CP, with no significant difference between them. Although the highest FBW was recorded in fish reared at 30 °C with 25% CP, this increase was not statistically significant. Similarly, this trend was observed in FCR, SGR, and FT, although the results slightly favor rearing at 28 °C with 25% CP.

Leptin plays a crucial role in nutritional status by regulating energy balance through reduced FI [44, 45]. Studies have shown a positive correlation between leptin and mesenteric somatic index, body lipid content, and growth performance, highlighting its role in managing lipid content for energy balance in fish [46–48]. Fabp, as lipid chaperones, are vital in lipid metabolism and transportation [49, 50]. The interaction between Fabp and leptin is key to modulating lipid metabolism [51]. In terms of the experimental results, there were no significant differences in hepatic leptin expression across groups. However, there were notable increases in hepatic *fabp* expression in fish given a 30% CP and raised at temperatures of 26 °C and 30 °C compared to those at 28 °C with a 25% CP. Additionally, similar to leptin expression, FI did not significantly change among groups. These findings suggest that both temperature and dietary protein content may have limited or no effect on these parameters.

Similarly, just as there were increases in hepatic *fabp* expression observed in fish given a 30% CP, there was also a notable rise in serum triglycerides among fish given 30% CP, especially those reared at 30 °C and 26 °C. This phenomenon can be attributed to the documented potential positive correlation between *FABP* expression and triglyceride levels [52, 53], considering that teleost fish tend to predominantly store lipids as triglycerides [54].

Adjusting dietary protein levels is crucial for optimizing immune and antioxidant systems, enhancing resistance against stressors and disease agents [13, 55, 56]. Research shows that dietary protein levels influence intestinal microbiota, intestinal barriers, and overall fish health [55, 57]. Our findings indicate that fish receiving 30% CP showed increased globulin levels within the same temperature group, with the best results observed at 28 °C regardless of their CP% and at 30 °C with 30% CP. Additionally, the highest serum IgM levels were observed at 26 °C with 30% CP, followed by 30 °C with 20% CP and 28 °C with 25% CP. This collectively suggests a positive correlation between dietary protein content and immunoglobulin levels.

Further supporting this, hepatic expression of *muc* increased across all groups, with a significant rise noted in fish at 30 °C with 30% CP compared to those at 28 °C with 25% CP. Hepatic expression of *pept1* and *lyz* also increased, particularly in fish raised at 30 °C with 30% CP. Mucin, an *O*-glycosylated glycoprotein, plays a defensive role against pathogens, and its intestinal expression is highly influenced by nutritional factors [58, 59]. Pept1 is involved in immune function and animal protein nutrition, optimizing amino acid absorption [60–63]. Lyz plays a critical role in the body’s first line of defense against pathogens [64, 65].

Given the strong connection between immune and antioxidant responses, enzymes like SOD, CAT, and GPX play a crucial role in protecting the body from oxidative stress, thereby safeguarding it from damage. They also have regulatory interactions with the immune response [66, 67]. It is highly proposed that their moderate levels and enhancement can significantly boost the body’s resistance and defense against stress, diseases, and maintain normal homeostasis [68, 69]. These enzymes shield the body from excessive reactive oxygen species that cause cellular damage [70].

In comparisons between different conditions, we observed notable downregulation of *sod* expression in fish at 28 °C with 30% CP, significant *cat* upregulation in fish at 30 °C with 25% CP, and a significant increase in *gpx* expression in fish at 28 °C with 20% CP, compared to those at 28 °C with 25% CP. However, serum levels of SOD and CAT were consistently good at 28 °C with either 25% or 30% CP, although the highest activities were recorded in fish raised at 30 °C with 25% CP. When considering ALT and AST results, these suggest the best antioxidative performance in groups at 28 °C regardless of CP% and at 30 °C with 25% CP.

## Conclusion

The study revealed a substantial influence of dietary protein levels and water temperature, as well as their interaction, on various parameters. Specifically, protein levels of 25% and 30%, along with temperatures of 28 °C and 30 °C, demonstrated favorable outcomes, particularly favoring the combination of 28 °C with 25% protein. This particular group exhibited strong performance in terms of growth, blood chemistry, and immune and antioxidant functions. Future research endeavors should encompass a broader range of rearing conditions and feeding patterns, comparing different factors to determine the optimal conditions for achieving peak performance in Nile tilapia across various life stages.

### Supplementary Information

Supplementary Material 1.

## Data Availability

The datasets produced and analyzed in this study can be obtained from the co-corresponding author upon a reasonable request.
