# Supplementary material for: Interactive effects of water temperature and dietary protein on Nile tilapia: growth, immunity, and physiological health
Source: BMC Vet Res. 2024 Aug 7;20:349. doi: 10.1186/s12917-024-04198-2 (PMC11304609; doi:10.1186/s12917-024-04198-2)
Supplement: Supplementary file 1 — Supplementary Material 1. [file 12917_2024_4198_MOESM1_ESM.docx]

**Additional File 1**

**Supplementary Table 1.** Composition and chemical analysis of the experimental diets (on dry matter basis); adopted from Hamed et al. (2021).

| **Ingredients composition of experimental diets** | **20% CP** | **25% CP** | **30% CP** |
| --- | --- | --- | --- |
| Fish meal (60% CP) | 2.0 | 2.5 | 3.2 |
| Soybean meal | 23.0 | 31.8 | 36.5 |
| Corn gluten | 2.5 | 4.0 | 8.0 |
| Yellow corn | 18.3 | 18.0 | 12 |
| Wheat middling's | 30.5 | 25.5 | 22.5 |
| Poultry by products meal | 0.0 | 2.5 | 4.0 |
| Rice bran | 19 | 11.4 | 8 |
| soy oil + rapeseed oil | 1.0 | 1.3 | 2.0 |
| Mono calcium phosphate | 0.9 | 0.6 | 0.6 |
| Common salt | 1.0 | 1.0 | 0.5 |
| Calcium carbonate | 2.0 | 1.2 | 0.5 |
| Pre-mix^a^ | 2 | 2 | 2 |
| **Chemical analysis (%)** | | | |
| Dry matter (DM %) | 90 | 90 | 90 |
| Metabolizable energy (kcal/kg) | 2,650 | 2,700 | 2,900 |
| Crude protein (CP %) | 20 | 25 | 30 |
| Ether extract (EE %) | 6.20 | 5.50 | 6.02 |
| Crude fiber (CF %) | 6.80 | 5.90 | 4.95 |
| Ash % | 5.3 | 5.6 | 5.1 |
| Available Phosphorus % | 0.37 | 0.39 | 0.4 |
| Calcium % | 1.2 | 1.1 | 0.99 |

^a^ Each 1 kg premix contains: Vit. A 4.8 I.U.; Vit. D2 0.8 I. U; Vit E, 4.0 g; Vit. K, 0.8 g; Vit B, 0.49, Vit. B2, 1.6 g; Vit. B6, 0.6 g; Vit. B12, 4 mg; Pantothenic acid 49 g; Nicotinic acid 8 g; Folic acid, 400 mg; Biotin, 20 mg; Choline chloride, 200 mg; Copper, 4.0 g; Iodine,0.4 g; Iron, 12 mg; Manganese, 22 g; Zinc 22 g and Selenium 0.04 g.

**References**

Hamed SA, Abou-Elnaga A, Salah AS, Abdel-Hay A-HM, Zayed MM, Soliman T, Mohamed RA (2021) Effect of water temperature, feeding frequency, and protein percent in the diet on water quality, growth and behavior of Nile tilapia *Oreochromis niloticus* (Linnaeus, 1758). J Appl Ichthyol 37:462–473. doi:10.1111/jai.14193
